# *Salmonella enterica* Prophage Sequence Profiles Reflect Genome Diversity and Can Be Used for High Discrimination Subtyping

**DOI:** 10.3389/fmicb.2018.00836

**Published:** 2018-05-04

**Authors:** Walid Mottawea, Marc-Olivier Duceppe, Andrée A. Dupras, Valentine Usongo, Julie Jeukens, Luca Freschi, Jean-Guillaume Emond-Rheault, Jeremie Hamel, Irena Kukavica-Ibrulj, Brian Boyle, Alexander Gill, Elton Burnett, Eelco Franz, Gitanjali Arya, Joel T. Weadge, Samantha Gruenheid, Martin Wiedmann, Hongsheng Huang, France Daigle, Sylvain Moineau, Sadjia Bekal, Roger C. Levesque, Lawrence D. Goodridge, Dele Ogunremi

**Affiliations:** ^1^Department of Food Science and Agricultural Chemistry, McGill University, Ste Anne de Bellevue, QC, Canada; ^2^Department of Microbiology and Immunology, Faculty of Pharmacy, Mansoura University, Mansoura, Egypt; ^3^Ottawa Laboratory Fallowfield, Canadian Food Inspection Agency, Ottawa, ON, Canada; ^4^Laboratoire de Santé Publique du Québec, Institut National de Santé Publique due Québec, Ste Anne de Bellevue, QC, Canada; ^5^Institut de Biologie Intégrative et des Systèmes, Université Laval, Québec City, QC, Canada; ^6^Health Canada, Bureau of Microbial Hazards, Ottawa, ON, Canada; ^7^Institute of Parasitology, McGill University, Montreal, QC, Canada; ^8^Centre for Infectious Disease Control, National Institute for Public Health and the Environment, Bilthoven, Netherlands; ^9^National Microbiology Laboratory, Public Health Agency of Canada, Guelph, ON, Canada; ^10^Department of Biology, Wilfrid Laurier University, Waterloo, ON, Canada; ^11^Department of Microbiology and Immunology, McGill University, Montreal, QC, Canada; ^12^Department of Food Science, Cornell University, Ithaca, NY, United States; ^13^Département de Microbiologie, Infectiologie et Immunologie, Université de Montréal, Montreal, QC, Canada; ^14^Département de Biochimie, de Microbiologie et de Bioinformatique, Université Laval, Québec City, QC, Canada

**Keywords:** *Salmonella*, prophage sequence typing, genome diversity, outbreaks, Enteritidis

## Abstract

Non-typhoidal *Salmonella* is a leading cause of foodborne illness worldwide. Prompt and accurate identification of the sources of *Salmonella* responsible for disease outbreaks is crucial to minimize infections and eliminate ongoing sources of contamination. Current subtyping tools including single nucleotide polymorphism (SNP) typing may be inadequate, in some instances, to provide the required discrimination among epidemiologically unrelated *Salmonella* strains. Prophage genes represent the majority of the accessory genes in bacteria genomes and have potential to be used as high discrimination markers in *Salmonella*. In this study, the prophage sequence diversity in different *Salmonella* serovars and genetically related strains was investigated. Using whole genome sequences of 1,760 isolates of *S. enterica* representing 151 *Salmonella* serovars and 66 closely related bacteria, prophage sequences were identified from assembled contigs using PHASTER. We detected 154 different prophages in *S. enterica* genomes. Prophage sequences were highly variable among *S. enterica* serovars with a median ± interquartile range (IQR) of 5 ± 3 prophage regions per genome. While some prophage sequences were highly conserved among the strains of specific serovars, few regions were lineage specific. Therefore, strains belonging to each serovar could be clustered separately based on their prophage content. Analysis of *S*. Enteritidis isolates from seven outbreaks generated distinct prophage profiles for each outbreak. Taken altogether, the diversity of the prophage sequences correlates with genome diversity. Prophage repertoires provide an additional marker for differentiating *S. enterica* subtypes during foodborne outbreaks.

## Introduction

*Salmonella* is a genus of Gram-negative bacteria composed of two species: *Salmonella bongori* and *Salmonella enterica*. The latter is divided into six subspecies and one of these subspecies, *S. enterica* subsp. *enterica* is divided into more than 2,500 serovars based on the surface antigens (Grimont and Weill, 2007). *S. enterica* includes all *Salmonella* of medical importance; these are divided into typhoidal serovars (Typhi and Paratyphi A, B, and C) and thousands of non-typhoidal serovars (NTS). Human infection with non-typhoidal *Salmonella* strains typically results in a self-limiting enterocolitis known as salmonellosis. The World Health Organization (WHO) estimated that approximately 180 million cases of salmonellosis occur in people every year with 298,000 deaths (WHO, 2016).

Salmonellosis typically results from consumption of contaminated food or water.

High discrimination subtyping of isolates is essential to link outbreak cases and for source tracking, so that intervention to prevent further illnesses may be made. This can be challenging for *S. enterica*, for though it is serologically diverse it is a relatively clonal pathogen and the majority of reported cases of salmonellosis can be attributed to a small group of serovars. In Canada, 75% of reported cases of salmonellosis are attributed to 10 serovars, with 57% of cases attributed to only 3 serovars: *S*. Enteritidis (30%), *S*. Heidelberg (15%), and *S*. Typhimurium (12%) (Public Health Agency of Canada [PHAC], 2002). Consequently, conventional *Salmonella* sub-typing schemes, including pulsed field gel electrophoresis (PFGE), phage typing and Multiple Locus Variable Number Tandem Repeat Analysis (MLVA) may lack the level of discrimination required to support effective public health decision making (Cooke et al., 2007; Feasey et al., 2012). Current whole genome sequencing (WGS)-based technologies can provide accurate and effective tools for the rapid subtyping of foodborne *Salmonella* (Brüssow et al., 2004; Ashton et al., 2017). However, in cases where a clearer distinction is needed among many closely related strains, e.g., delineating different outbreak strains or separating outbreak and sporadic strains, current WGS methodologies do not consistently meet the required need. Therefore, developing a parallel WGS-based subtyping tool may improve the differentiation among highly clonal *Salmonella* isolates.

The pan-genome of a bacterial species consists of a core genome which is the collection of coding sequences present in all members of the species and an accessory genome made up of sequences that are not consistently present among all members. The core genome defines the common attributes of the species whereas the accessory genome affect strain fitness by encoding proteins that influence virulence, antigenic structure, antimicrobial resistance, and metabolic characteristics. The accessory genome can represent up to 10% of the typical *S. enterica* genome (Jacobsen et al., 2011). These sequences include mobile genetic elements such as prophages, plasmids, transposons, and insertion elements (Thomson et al., 2008). Prophage sequences are common in the genome of *Salmonella* and their potential as molecular markers of genomic diversity has been previously investigated (Brüssow et al., 2004; Cooke et al., 2007; Feasey et al., 2012). For example, prophage sequences were used to discriminate between different field isolates of *S*. Typhimurium (Cooke et al., 2007). Moreover, two African and two global epidemic clades of *S*. Enteritidis were recently discriminated by their prophage repertoire (Feasey et al., 2016). Others have used a single prophage gene, the prophage integrase, to demonstrate genomic diversity in *S. enterica* (Colavecchio et al., 2017a). However, no single gene is likely to provide a comprehensive enough information for taxonomic analyses (Rohwer and Edwards, 2002) and is likely applicable only within a narrow spectrum of diversity such as within the same serovar of *Salmonella* (Colavecchio et al., 2017a).

Prophage sequences have been shown to encode virulence factors, toxins, and antimicrobial resistance genes (Brüssow et al., 2004; Colavecchio et al., 2017b) all of which contribute to diversity of strains. Therefore, we postulated that prophage features may be one of the principal drivers of diversification among and within serovars of *Salmonella*. To that end, we aimed to conduct a comprehensive characterization of prophage diversity in 151 serovars of *S. enterica* and to test the performance of the whole prophage repertoire as a measure of the host organism diversity. We tested the discriminative ability of this approach among *S. enterica* serovars, closely related isolates of the same serovar from different outbreaks and finally between predominant *S. enterica* serovars and other closely related members of the family Enterobacteriaceae including *Proteus, Hafnia*, and *Citrobacter*, which are sometimes misidentified as *Salmonella* isolates.

## Materials and methods

### Bacterial strains and whole genome sequencing

A set of 1,826 bacterial strains was used in the current study. These strains included *S. enterica* isolates (*n* = 1,760) from 151 serovars (Table 1), *S. bongori* isolates (*n* = 2), *Citrobacter amalonaticus* (*n* = 6), *C. freundii* (*n* = 14), *C. braakii* (*n* = 5), *C. koseri* (*n* = 2), *C. farmeri* (*n* = 1), *C. werkmanii* (*n* = 1), other *Citrobacter spp*. (*n* = 9), *Hafnia alvei* (*n* = 11), *Proteus mirabilis* (*n* = 14), and *P. vulgaris* (*n* = 1). The strains were isolated from different sources: 527 from humans; 217 from domestic and wild animals; 117 from poultry; 267 from environmental samples; 48 from fish; 7 from dairies; 35 from fruits and vegetables; 17 from nuts or seeds, 15 from animal feed, and 576 from undetermined or unspecified food sources (Supplementary Table 1). The isolates originated from 27 countries and the Middle East. Although the majority of isolates came from Canada (*n* = 877) and the United States (*n* = 551), sizeable numbers were sourced from the Netherlands (*n* = 53), Jamaica (*n* = 40), France (*n* = 32), Switzerland (*n* = 13), and Scotland (*n* = 12). Fewer than 10 isolates came from each of the remaining 20 countries and the Middle East as shown in Supplementary Table 1. All *S. enterica* isolates were typed serologically by the classical Kauffman-White procedure in use by *Salmonella* reference laboratories around the world. Genomes of these isolates were sequenced at the EcoGenomics Analysis Platform of the Institute for Integrative and Systems Biology (IBIS; Université Laval) as previously described (Emond-Rheault et al., 2017). Briefly, 300 bp paired-end reads were generated on an Illumina MiSeq from large insert libraries to a sequencing depth of approximately 45X coverage on average per strain. The raw reads, in addition to the genomic and metadata, were submitted to the *Salmonella* Foodborne Syst-OMICS database (SalFoS; https://salfos.ibis.ulaval.ca/; Emond-Rheault et al., 2017).

**Table 1:** *Salmonella enterica* serovars and non*-Salmonella* species with the corresponding number of genomes investigated in the current study.

**Serovar**	**#**	**Serovar**	**#**	**Serovar**	**#**	**Serovar**	**#**	**Serovar**	**#**
Aarhus	1	Chailey	1	Hadar	9	Manhattan	10	Roodepoort	1
Abaetetuba	1	Chester	10	Haifa	6	Mbandaka	11	Rubislaw	10
Aberdeen	6	Chingola	1	Hartford	14	Meleagridis	2	Saintpaul	22
Adelaide	8	Choleraesuis	11	Havana	10	Miami	10	Sandiego	11
Agbeni	1	Corvallis	7	Heidelberg	201	Minnesota	1	Schwarzengrund	12
Ago	1	Cotham	1	Hull	1	Mississipi	14	Sendai	2
Agona	17	Cremieu	1	Hvittingfoss	10	Monschaui	1	Senftenberg	25
Alachua	9	Cubana	1	Ibadan	1	Montevideo	11	Singapore	4
Albany	9	Daytona	2	Indiana	1	Muenchen	20	Solt	1
Amager	1	Decatur	1	Idikan	1	Muenster	12	Stanley	12
Anatum	21	Derby	12	Infantis	21	Nessziona	4	Stanleyville	4
Arechavaleta	2	Dessau	3	Irumu	1	Newport	58	Tado	1
Arizonae	3	Dublin	11	Isangi	1	Nyanza	1	Taiping	1
Ball	1	Duesseldorf	1	Java	4	Ohio	10	Taksony	1
Banana	1	Duisburg	1	Javiana	13	Oranienburg	11	Telelkebir	9
Bardo	2	Durban	2	Johannesburg	1	Orion	2	Tenessee	16
Bareilly	12	Ealing	1	Kentucky	12	Oslo	5	Thompson	24
Barranquilla	1	Eastbourne	10	Kiambu	11	Panama	7	Tornow	1
Bergen	1	Elisabethville	1	Kintambo	1	ParatyphiA	12	Typhi	20
Berta	13	Emek	1	Kisarawe	2	ParatyphiB	40	Typhimurium	148
Blockley	10	Enteritidis	208	Kottbus	5	ParatyphiC	2	Typhisuis	2
Bonariensis	1	Falkensee	1	Kouka	2	Pasing	1	Tyresoe	1
Bovismorbificans	9	Freetown	1	Larochelle	6	Pomona	8	Uganda	9
Braenderup	18	Fresno	1	Lille	1	Poona	12	Virchow	10
Brandenburg	9	Gallinarum	2	Litchfield	8	Pullorum	2	Wandsworth	1
Bredeney	10	Gaminara	8	Liverpool	8	Putten	2	Weltevreden	8
Broughton	1	Georgia	1	London	10	Reading	8	Wentworth	1
Canada	1	Give	8	Loubomo	1	Richmond	1	Westhampton	1
Casablanca	1	Glostrup	1	Luciana	2	Rissen	9	Weston	1
Cerro	10	Godesberg	1	Luckenwalde	1	Rissen-Ardwick	1	Wien	3
Worthington	1	*S. bongori*	2	*C.amalonaticus*	6	*C. freundii*	14	*C. braakii*	5
*C. koseri*	2	*C. farmeri*	1	*C. werkmanii*	1	*Citrobacter spp*.	9	*Hafnia alvei*	11
*Proteus mirabilis*	14	*P. vulgaris*	1						

### Prophage sequence detection

Even though the *Salmonella* genomes used in this study were assembled previously using A5 (Tritt et al., 2012; Coil et al., 2015) at a high level of quality (Emond-Rheault et al., 2017), we decided to reassemble the raw data using SPAdes genome assembler version 3.10.1 with updates available at: http://bioinf.spbau.ru/en/spades (Bankevich et al., 2012). The raw paired-end Illumina reads were trimmed to a minimum PHRED quality score of Q10 from the 3′ end using the BBDuk tool (http://jgi.doe.gov/data-and-tools/bbtools/). Trimmed reads shorter than 64 bases were discarded and the remainders were merged using BBMerge (http://jgi.doe.gov/data-and-tools/bbtools/). Both paired and unpaired reads were kept for *de novo* assembly, which was performed using SPAdes genome assembler 3.10.1 algorithm (Bankevich et al., 2012). The quality of each assembled genome was assessed following analysis with the QUAST software (Gurevich et al., 2013) and all were satisfactory based on the number of contigs, size of the largest contig, number of bases in the assembly, and NG_50_ (data not shown). Prophage sequences within the assembled contigs of each genome were identified with the PHAge Search Tool Enhanced Release (PHASTER; Zhou et al., 2011; Arndt et al., 2016). All contigs <2 Kb were discarded before submitting the assembly to PHASTER, as PHASTER processes only contigs of length >= 2 kb.

### Prophage sequence typing

We developed a prophage sequence typing procedure by clustering the identified prophages sequences present in the genomes. The prophage regions identified by PHASTER were extracted from each genome and every sequence was renamed by adding the sample name to the sequence header. All identified prophage regions from the strains analyzed were clustered using CD–HIT-EST (Fu et al., 2012) based on 90% identity and 90% prophage sequence length coverage. Increasing the stringency to identity and sequence coverage of 95, 99, and 100% did not improve the clustering efficiency at the serovar level (data not shown), hence the 90% threshold was selected. Due to the high genomic clonality of *S*. Enteritidis isolates we used 100% sequence identity and alignment coverage for comparing the prophage profiles of *S*. Enteritidis outbreak isolates. The representative sequence of each cluster generated by CD–HIT-EST was used to determine the prophage identity of that cluster by local alignment of the sequences against the PHASTER prophage virus database using BLASTX (Altschul et al., 1990). A prophage cluster matrix table was then constructed from the CD–HIT-EST and BLASTX results showing the prophage cluster number, the length and the identity of each prophage sequence detected in every analyzed genome (Supplementary Table 2).

### Prophage diversity and composition analyses

The prophage matrix table was fed to Quantitative Insights Into Microbial Ecology (QIIME) analytical platform (Caporaso et al., 2010) to determine the diversity of prophage repertoire within (alpha diversity) and among (beta diversity) the different strains in addition to prophage taxonomy summarization. To test the variability of prophage profiles between two or more groups of samples, the diversity of the identified prophage clusters was used to calculate the Bray-Curtis distances among different strains and the generated distances were employed in Unweighted Pair Group Method with Arithmetic mean (UPGMA) clustering. The generated UPGMA tree files were exported to the Interactive Tree of Life (iTOL) software (Letunic and Bork, 2011) for viewing and editing. The identified RE-2010 sequences in *S*. Enteritidis genomes from known outbreaks (*n* = 111) were extracted from PHASTER results and aligned with Multiple Alignment using Fast Fourier Transform (MAFFT; Katoh and Standley, 2013) tool from Galaxy platform (https://usegalaxy.org) and the alignment tree file was uploaded to iTOL platform for editing. We conducted an analysis of similarity (ANOSIM) on the prophage sequence profiles using QIIME where the *p*-value was calculated using 999 permutations; *p*-value <0.05 was considered significant. ANOSIM generates *R*-value that lies between 0 and +1 where the closer the value to +1 the higher the dissimilarity of the prophage profiles among the compared groups of isolates (i.e., among different serovars, outbreaks, or species) (Mottawea et al., 2016).

Furthermore, a supervised learning serovar classification using QIIME was conducted by training the supervised random forests classifier with the prophage clusters as predictors and the serovar name as the class labels. This supervised learning analysis generated a ranking of prophage clusters by importance, estimated the classifier out-of-bag error, and predicted the serovar label in addition to the probabilities of classification.

## Results

### Prophage sequence diversity in *S. enterica* serovars

**Figure 1:**
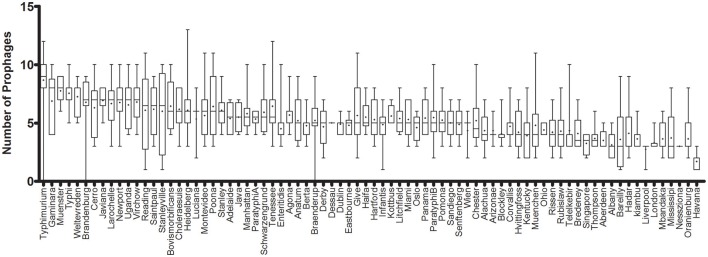
Prophage sequence diversity among different *Salmonella enterica* serovars. Only serovars with more than two isolates are represented. The columns are sorted in a decreasing order of the median number of prophages per serovar. The middle line represents the median and + represents the mean prophage number per genome, while the bar indicates the range of prophage numbers.

The prophage sequence diversity was characterized among the whole genome sequences of 1,760 *S. enterica* isolates with 86% or 1,508 isolates belonging to 151 serovars, while the serovar designations of 14% or 252 isolates were unknown. Overall, 11,297 discrete prophage sequences were identified among all the total genomes, with a median of 5 and interquartile range (IQR) of three prophage sequences per isolate. These sequences were grouped into 3,126 clusters based on 90% sequence identity and 90% long sequence coverage. No prophage sequence was detected in 3 *S*. *enterica* strains belonging to serovars Braenderup, Brandenburg and Typhimurium, despite a genome sequence coverage of 16, 22, and 19X, respectively. We identified only one prophage sequence in 12 strains (4 Havana, 3 Bareilly, and 1 strain from each of Gallinarum, Infantis, Liverpool, Putten, and Stanleyville serovars). In contrast, the highest number of prophage sequences in one genome (*n* = 15) was detected in a strain belonging to serovar Kisarawe. Prophage sequences were most prevalent in *S*. Typhimurium (148 strains) (Median ± IQR of 9 ± 2 prophages per isolate). The genomes of the other two common serovars; *S*. Enteritidis (208 strains) and *S*. Heidelberg (201 strains) carried 5 ± 1 and 6 ± 2 prophage sequences per isolate, respectively. *S*. Havana serovar (10 strains) had the fewest prophages (2 ± 1) (Figure 1 and Supplementary Table 3).

### Prophage sequence profiles in *S. enterica* genomes

**Figure 2:**
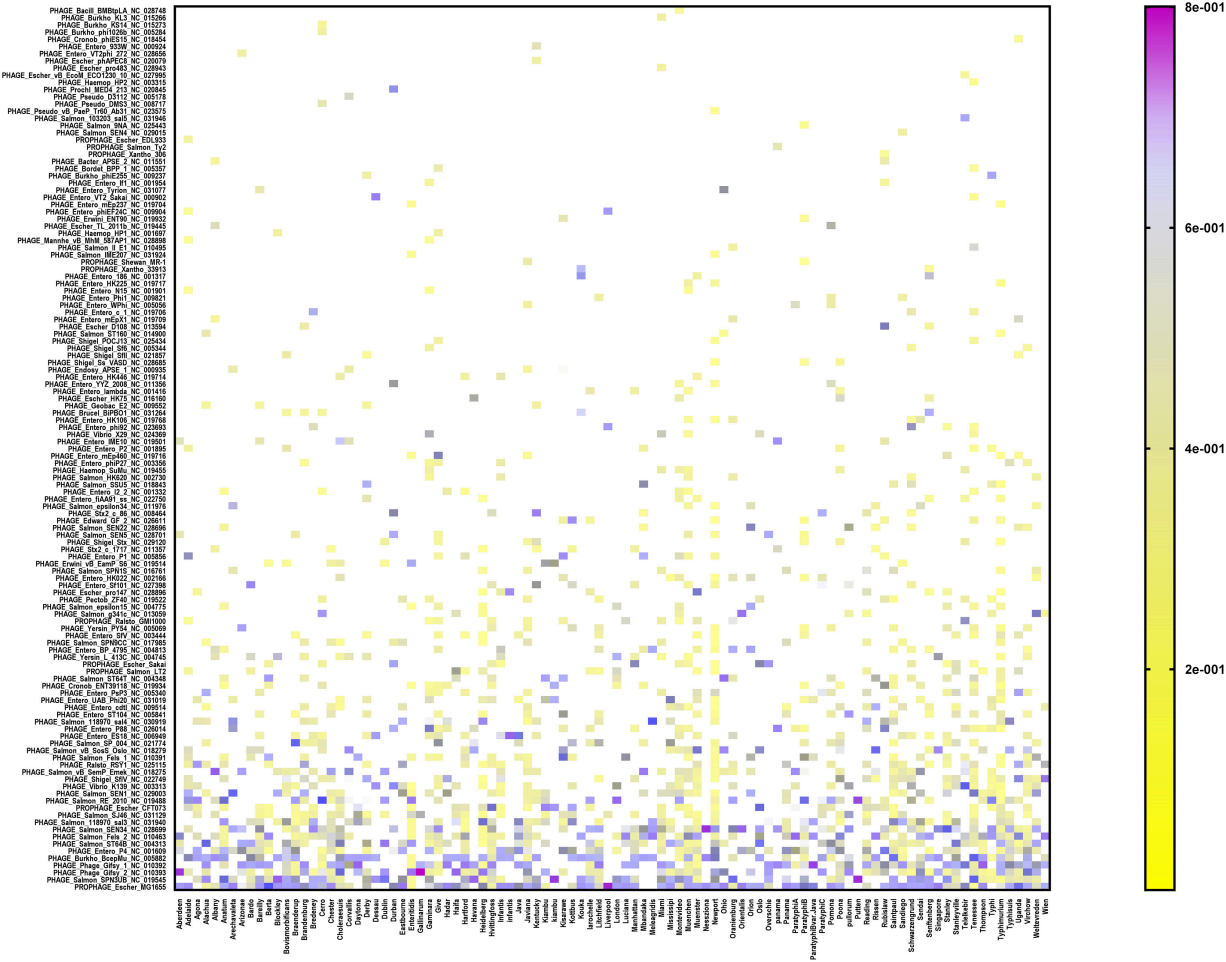
Heat map showing the prophage repertoire in different *Salmonella enterica* serovars. The color scale denotes the relative length of each prophage compared to the collective prophage sequences detected in each serovar with the violet color denoting maximum contribution of one prophage (i.e., up to 80%). Prophages are sorted according to their prevalence among different serovars in a decreasing order from the bottom to the top of the figure.

In order to assign a prophage identity to every prophage sequence detected, the CD–HIT-identified reference sequence from each prophage cluster (see under Prophage sequence clustering, above) was aligned against the PHASTER prophages/virus database (last updated on Feb 9, 2017), using the BLASTX tool. Among the 151 serovars (*n* = 1,508 isolates), we detected 154 different prophages (Figure 2 and Supplementary Table 3). The most prevalent prophages identified among our cohort of *S. enterica* serovars were *Escherichia* phage MG1655 which was present in 82 out of 151 serovars, PHAGE *Salmonella* phage SPN3UB in 63 serovars, Phage Gifsy 2 in 61 serovars as well as Phage Gifsy 1 and *Burkholderia* phage BcepMu in 60 serovars (Figure 2 and Supplementary Table 3). We identified 41 different prophages in *S*. Typhimurium isolates (*n* = 144) which included highly prevalent prophages such as *Burkholderia* phage BcepMu (99.3% of the isolates), Gifsy-2 (95.8%), Gifsy-1 (91.7%), *Salmonella* phage SJ46 (70.1%), *Enterobacteria* phage SfV (65.3%), *Escherichia* phage EB49 (CFT073; 61.8%), *Escherichia* phage MG1655 (59.7%), and *Salmonella* phage ST64B (50.7%). In the case of *S*. Enteritidis (*n* = 208), we identified 21 different prophages, while the serovar Heidelberg (*n* = 196) harbored 26 different prophages. The predominant prophages in *S*. Enteritidis were *Erwinia* phage vB EamP S6 (96.2%), Gifsy-2 (95.8%), *Salmonella* phage SJ46 (88.5%), *Salmonella* phage RE-2010 (81.3%), and *Salmonella* phage ST64B (61.5%), while seven prophages were the most common among Heidelberg isolates: *Burkholderia* phage BcepMu (100%), Gifsy-2 (97.4%), *Salmonella* phage 118970 sal4 (92.3%), *Salmonella* phage 118970 sal3 (79.6%), *Escherichia* phage MG1655 (64.8%), *Enterobacteria* phage UAB Phi20 (63.3%), and *Escherichia* phage EB49 (53.6%).

### Exploiting prophage sequence diversity for *Salmonella enterica* subtyping

**Figure 3:**
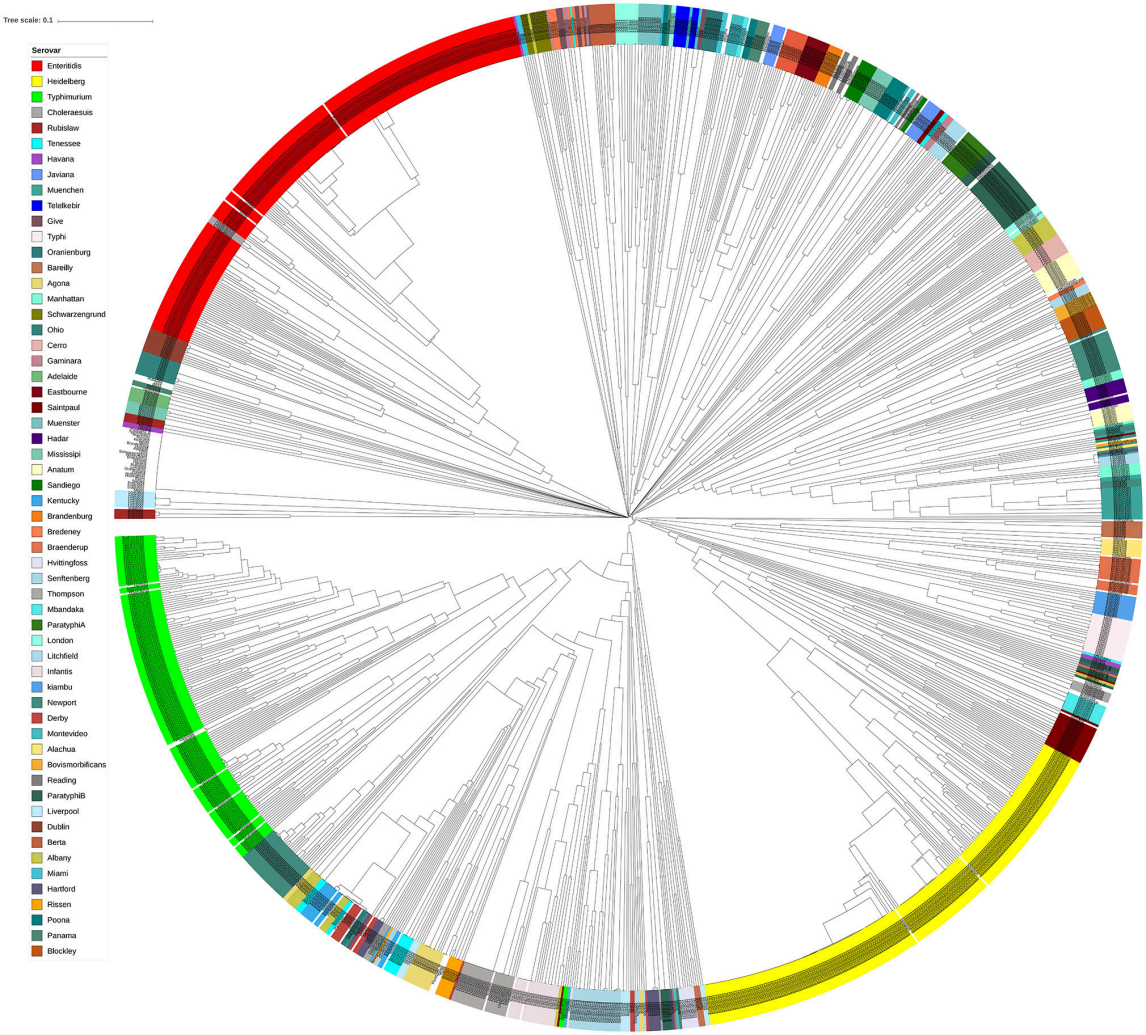
Isolates of different *Salmonella* serovars clustered separately based on their prophage sequence diversity. Bray-Curtis distances among 1,427 *Salmonella* isolates were calculated based on genomic prophage sequences diversity and were applied in Unweighted Pair Group Method with arithmetic mean (UPGMA) hierarchical clustering using Quantitative Insights Into Microbial Ecology (QIIME) pipeline. Analysis of Similarity (ANOSIM) *R* = 0.884; *p* = 0.001.

In order to assess the performance of genomic prophage sequence diversity as a discriminatory marker for the different *Salmonella* serovars, we calculated Bray-Curtis distance among the prophage profiles of different strains and the generated distance matrix was used to construct a phylogenetic tree of the strains. In order to reduce the bias generated by low isolate number and prophages that may be detected due to analytical errors, this analysis included those serovars that had eight or more *Salmonella* strains (*n* = 1,350 isolates) and by using only prophage clusters detected in at least four strains. We found that strains within each serovar clustered separately from strains of other serovars as indicated by the constructed phylogenetic tree (Figure 3). Clustering of serovars based on prophages was found to be statistically significant following ANOSIM evaluation (*R* = 0.88, *p* = 0.001). In order to test the ability to predict the *S. enterica* serovar from the prophage sequences, we also conducted a supervised machine learning algorithm using random forests classifier and out-of-the-bag prediction to estimate the generalization error. The estimated generalization error was 0.15, while the baseline error (for random guessing) was 0.85 and the ratio of baseline error to the observed error was 5.38. The model confusion matrix, cv_probabilities and mislabelling matrix are detailed in Supplementary Tables 4–6. In general, these results indicate a significant correlation between the identified prophage profiles and the genome diversity of different *S. enterica* serovars.

### Prophage sequence diversity differentiates *Salmonella enterica* serovar enteritidis outbreak isolates

**Figure 4:**
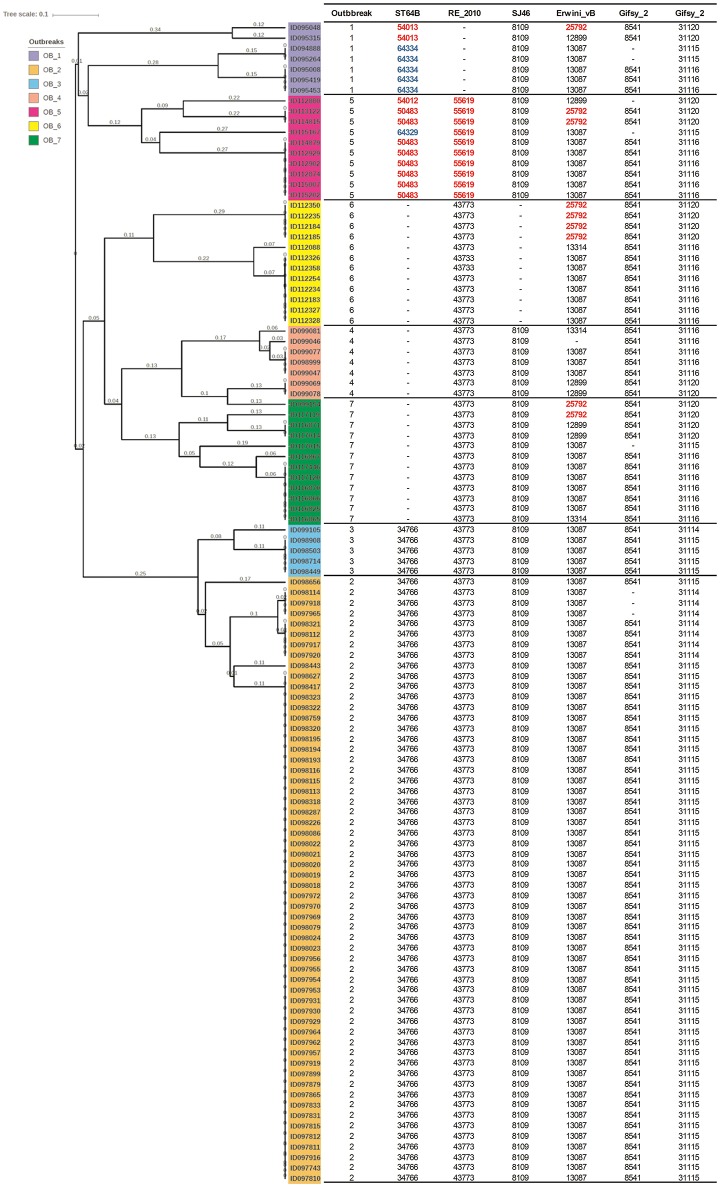
Prophage sequence profiles discriminated among *Salmonella* Enteritidis isolates from seven outbreaks. Bray-Curtis distances among *S*. Enteritidis strains obtained from seven outbreaks (*n* = 111) were calculated based on prophage sequence diversity and were applied in Unweighted Pair Group Method with Arithmatic Mean (UPGMA) hierarchical clustering using Quantitative Insights Into Microbial Ecology (QIIME) pipeline. The numbers in the table refer to the length of the prophage region detected, while the red and blue colors indicate different length variants of the same prophage. Numbers on the tree branches refer to the branch length. Analysis of Similarity (ANOSIM) *R* = 0.98; *p* = 0.001.

**Figure 5:**
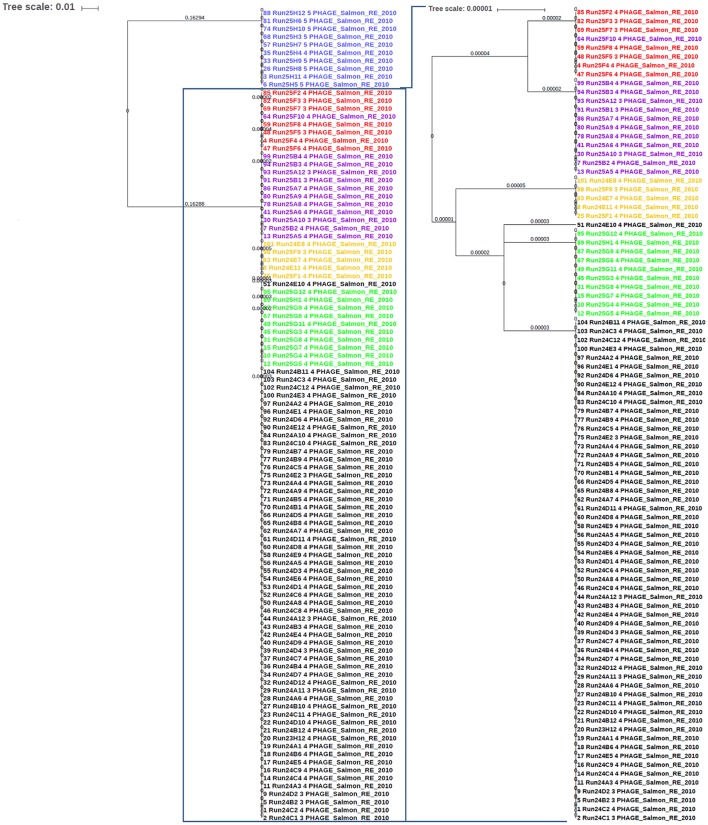
The sequence of phage RE-2010 distinguished among *Salmonella* Enteritidis isolates from different outbreaks. The identified RE-2010 sequences in *S*. Enteritidis genomes involved in known outbreaks (*n* = 111) were extracted from PHASTER results and aligned with Multiple Alignment using Fast Fourier Transform (MAFFT) tool from Galaxy platform (https://usegalaxy.org). The alignment tree was exported to the Interactive Tree of Life (iTOL) software for viewing and editing. Numbers on branches represent the corresponding branch length. Blue color represents strains of outbreak 6; Red for outbreak 4; Purple for outbreak 8, Orange for outbreak 3, Green for outbreak 7, and Black for outbreak 2.

*Salmonella enterica* serovars are characterized by a high genetic clonality. For this reason, conventional typing methods such as PFGE cannot reliably differentiate between closely related strains (Bekal et al., 2016). Although typing by SNPs has been successfully used to differentiate strains of highly clonal serovars, such as *S*. Enteritidis (Ogunremi et al., 2014; Ashton et al., 2016) the high resolution does not always result in a separation of outbreak and sporadic strains. For these reasons, development of an accurate, parallel subtyping method should provide additional tools to discriminate bacterial strains. To this end, we investigated the correlation between prophage sequence profiles and the genome diversity among 111 *S*. Enteritidis strains isolated during seven outbreaks. A total of 607 prophage sequences was detected in the genomes of these strains. The identified prophage sequences were clustered based on 100% identity and sequence coverage. A UPGMA clustering tree was generated based on Bray-Curtis distances among the prophage profiles of these strains. We identified 43 prophage sequence clusters that belong to five different prophages in our dataset. The UPGMA clustering resulted in a complete separation of these strains into seven clusters with each cluster specific to an outbreak, except only one strain isolated from outbreak 7 that clustered with outbreak 4 (Figure 4; ANOSIM *R* = 0.98; *p* = 0.001). The large Gifsy-2 prophage fragment was consistently detected in all isolates (100%, fragment length = 31,114–31,120 bases). The small Gifsy-2 prophage fragment (8,541 bases) was also very prevalent (93%) but was missing in a few isolates. Similarly, prophage *Erwinia* phage vB was present in almost all the isolates (99%) either as a small (13,087 bases), or large (25,792 bases) fragment. On the other hand, four different sequence variants of prophage ST64B were detected in our Enteritidis dataset (Image 1): one variant was specific to outbreaks 2 and 3 (length = 34,766 bases), while the other three variants were distributed among the isolates of outbreaks 1 and 5 (lengths = 50,483, 54,013, and 64,334 bases). We did not detect phage ST64B sequence in strains from outbreaks 4, 6, and 7. Interestingly, the prophage RE2010 was detected as six different clusters (Figure 5): one specific for each of the outbreaks 2, 3, 4, 5, 6, and 7, whereas this prophage was not detected in outbreak 1 (Figure 4). Finally, we did not detect any sequence corresponding to the prophage SJ46 in outbreak 6 (Figure 4), but it was identified in other outbreaks with a length of 8,109 bases. These findings indicate that prophage sequence profiles distinguished among the different *S*. Enteritidis subtypes responsible for distinct foodborne outbreaks.

### Non-typhoidal *Salmonella* isolates harbour prophage sequence profiles distinct from other closely related enterobacteriaceae

**Figure 6:**
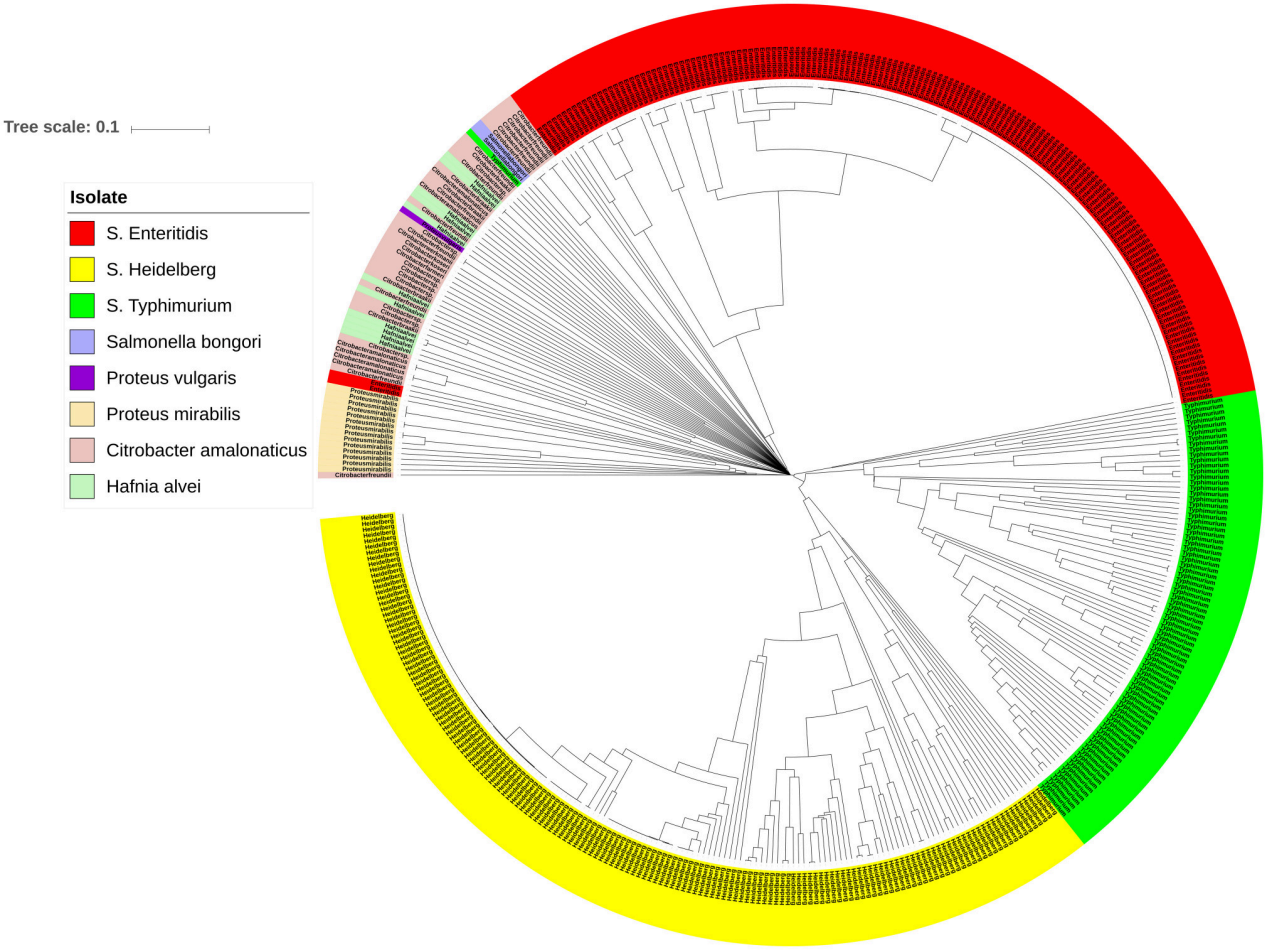
Prophage sequence profiles distinguished between *Salmonella enterica* and other related bacteria. Bray-Curtis distances among *Salmonella* and non-*Salmonella* isolates (*n* = 453) were calculated based on genomic prophage sequences diversity and were applied in Unweighted Pair Group Method with arithmetic mean (UPGMA) hierarchical clustering using Quantitative Insights Into Microbial Ecology (QIIME) pipeline. Analysis of Similarity (ANOSIM) *R* = 0.86; *p* = 0.001.

The bacterial set in this study included organisms commonly misidentified as *S. enterica* strains such as *Citrobacter, Proteus*, and *Hafnia*, as well as *S. bongori*. To investigate the genomic prophage sequence profiles in these pathogens as compared to *S. enterica*, we extracted the prophage clusters identified in these bacteria along with those identified in *S*. Enteritidis, *S*. Heidelberg and *S*. Typhimurium isolates, as these serovars represent the majority of our *S. enterica* set. UPGMA clustering showed that these organisms which can cause false positives clustered separately from *S*. Enteritidis, *S*. Heidelberg and *S*. Typhimurium isolates (Figure 6). ANOSIM *R*-value was 0.86 with *p*-value of 0.001. In contrast to *Salmonella* isolates, we observed that the prophage profiles of these bacteria were highly variable among strains analyzed with the exception of *P. mirabilis*, which were clustered together. This conservation among *P. mirabilis* isolates mainly arises from one prophage cluster identified as *Shewanella* phage 1/41 NC 025458 which was present in 13 out of the 14 isolates (Supplementary Table 7). These results revealed that the prophage sequences integrated in the genome of *S. enterica* isolates are distinct from those harbored by the genomes of other closely related bacteria of the family Enterobacteriaceae.

## Discussion

Bacteriophages appear to be significant drivers of the evolution of pathogenic bacteria including *S. enterica* (Figueroa-Bossi and Bossi, 1999; Figueroa-Bossi et al., 2001; Davies et al., 2016; Diard et al., 2017). Accordingly, prophage sequences constitute a significant component of *Salmonella* accessory genomes (Ogunremi et al., 2014; Owen et al., 2017) and have been reported to enable discrimination among closely related *Salmonella* lineages (Kingsley et al., 2009; Okoro et al., 2015; Colavecchio et al., 2017a; Owen et al., 2017). This discriminatory power of prophage sequences has been demonstrated also for two different serovars of *Streptococcus pyogenes* (Brüssow et al., 2004).

The current study reports a pipeline for the rapid identification of genomic prophage sequences in *Salmonella* and to use them to discriminate between different epidemiological categories and serovars. Previous efforts to characterize bacterial strains and distinguish one from another based on their prophage content have followed different approaches. Some studies have relied on descriptive methods that compare the number of prophages and their identity among a certain number of bacterial strains within specific taxonomic groups (Okoro et al., 2015; Zheng et al., 2017). In general, these studies focussed on prevalent and intact prophage regions while neglecting less predominant and incomplete ones. Some prophages exhibit sequence variants that are differentially integrated among distinct host organisms and serovars (Mmolawa et al., 2003; Bobay et al., 2013; Hiley et al., 2014; Zheng et al., 2017). For example, Bobay and coauthors have detected different prophage sequences in the whole genome of 20 strains of *S. enterica* and classified them based on their sequence similarity in order to understand prophage integration patterns and their genetic adaptation to host genomes (Bobay et al., 2013). These sequence variants may arise from modular exchange with other prophage DNA, mutation, adaptational shift, and/or drift (Brüssow et al., 2004; Boyd et al., 2012; Omer et al., 2017) and these changes can be expected to have evolutionary implications (Diard et al., 2017). To that end, prophage diversity within bacterial strains does not only rely on the presence or absence of a certain prophage, but also on the conservation of the prophage sequences. Few studies have targeted these sequence variations in certain phage genes to assess the diversity of both phages and their host genomes (Adriaenssens and Cowan, 2014; Colavecchio et al., 2017a). Since there is no universal phage gene (Rohwer and Edwards, 2002), relying on one gene will narrow the spectrum of the discriminative approach to phages that have this gene. Additionally, each prophage sequence feature represents a specific evolutionary event in the bacterial genome (Brüssow et al., 2004).

Here, we analyzed every sequence detected by PHASTER (Arndt et al., 2016) as a distinct operational unit that has its own discriminative weight among *Salmonella* strains. Moreover, we identified prophage sequence clusters based on the similarity and coverage lengths so that sequence variants are analyzed as distinct operational units. It should be noted that the performance of the current pipeline is limited by the sensitivity and the accuracy of the incorporated assembly and prophage detection tools. For example, we may lose some prophage regions because of improper sequence assembly. Furthermore, PHASTER appears to have the same sensitivity index (85.4%) as its predecessor, PHAST (Zhou et al., 2011). Therefore, the performance errors of the genome assembly and prophage detection tools involved in our pipeline could be reflected on the sensitivity and the prediction accuracy of our approach. Despite this potential limitation, the use of PHASTER proved to be adequately sensitive and the procedure outlined highly discriminative of epidemiologically unrelated strains of Enteritidis.

*Salmonella* populations are characterized by highly variable prophage profiles (Figueroa-Bossi et al., 2001; Brüssow et al., 2004; Owen et al., 2017). This is largely consistent with our finding that the prophage repertoires identified in this study are highly variable among different *Salmonella* serovars. The number of prophage sequences detected ranged from 1 to 15 regions per genome. Even within the same serovar, there is a high variability in prophage combinations between strains. For example, we detected a median of 9 ± 2 (IQR) prophages per genome in *S*. Typhimurium isolates. Eight of these prophages (*Burkholderia* phage BcepMu, Gifsy-2, Gifsy-1, *Salmonella* phage SJ46, *Enterobacteria* phage SfV, *Escherichia* phage EB49, *Escherichia* phage MG1655, *Salmonella* phage ST64B) are prevalent among *S*. Typhimurium genomes. Gifsy-2, Gifsy-1, and ST64B are known to be common among different *S*. Typhimurium lineages (Kingsley et al., 2009; Okoro et al., 2015; Owen et al., 2017). Certain prophages, such as Gifsy-2, are highly prevalent among different *Salmonella* serovars. We detected Gifsy-2 in 61 serovars of *S. enterica* and it is present in more than 95% of *S*. Enteritidis, *S*. Heidelberg and *S*. Typhimurium isolates. Still, Gifsy-2 exhibits three different sequence variants among the three serovars (Image 2). This result is consistent with Colavecchio et al. who reported that one gene of this prophage was sufficient to differentiate between *S*. Enteritidis and *S*. Heidelberg isolates (Colavecchio et al., 2017a). In addition to the genetic variants of common prophages, newly acquired prophages or lineage-specific prophages may contribute to strain divergence as shown recently with *S*. Newport (Zheng et al., 2017). In general, this variability in prophage sequence types illustrates the correlation between prophage repertoires and the genome diversity of different *Salmonella* serovars.

Foodborne Salmonellosis is an important concern for public health (Ziebell et al., 2017). To reduce the impact of outbreaks caused by foodborne pathogens, timely subtyping of isolates to incriminate sources of contamination is crucial. Conventional *Salmonella* typing methods such as PFGE, phage typing (PT), MLVA, and multiple amplification of phage locus typing (MAPLT) do not always provide the required level of discrimination (Assis et al., 2017; Ziebell et al., 2017). It was proposed previously that prophage composition could differentiate between *S*. Enteritidis subtypes during foodborne outbreaks (Ogunremi, 2013). Although prophage sequences are highly variable among *Salmonella* and can sometimes be transient and mobile (Owen et al., 2017), herein we have established that these repertoires correlate with outbreak strains of *Salmonella* with these results illustrate how prophage sequences can accurately distinguish between epidemiologically unrelated isolates. While Gifsy-2 prophage sequence is common among all the isolates from different outbreaks as noted by others (Colavecchio et al., 2017a), three other prophages (RE2010, ST64B, and SJ46) exhibit outbreak-specific sequences and profiles. Interestingly, a single prophage sequence (phage RE2010) was enough to differentiate between the investigated seven outbreaks. This finding supports a previous report on the contribution of RE2010 to separate *S*. Enteritidis isolates with the same PFGE pattern (Allard et al., 2013) but considerably expanded on previous observations by showing a common prophage profile among isolates recovered from each outbreak of *S*. Enteritidis, which distinguished the different outbreaks. Furthermore, prophages ST64B and SJ46 have been shown previously to be present as multiple variants among closely related *S. enterica* genotypes of the same serovar (Mmolawa et al., 2003; Hiley et al., 2014; Zheng et al., 2017). Therefore, we conclude that prophage diversity can serve to differentiate epidemiologically unrelated subtypes of *S*. Enteritidis. In addition, considering that single nucleotide polymorphism (SNP) in a 60-loci test can differentiate between unrelated *S*. Enteritidis (Ogunremi et al., 2014), detecting SNPs within the sequence of universally distributed prophages, such as Gifsy-2 or RE2010, may provide a synergistic mean of differentiating *S*. Enteritidis subtypes during foodborne outbreaks.

One difficulty in the detection of foodborne *Salmonella* is the misidentification of strains of *Citrobacter, Hafnia*, and *Proteus spp*. as *Salmonella* (Muldoon et al., 2007; Fang et al., 2009; Margot et al., 2013). The results of this study revealed that the prophage profiles in these false positive strains are completely different from those of *S. enterica* isolates. In contrast to *Salmonella* prophages, which are characterized by high degree of conservation within the same serovar, the prophage profiles of *Citrobacter* and *Hafnia* spp. are characterized by high degree of variability among strains. For *P. mirabilis*, only one prophage remnant sequence is common among its isolates, while the other sequences are highly variable. Therefore, it is clear that prophage sequences could provide specific *S. enterica* targets for development of inclusive and exclusive detection tools of foodborne *Salmonella* pathogens.

In conclusion, this study reports a new pipeline for the detection of prophage sequence diversity in *S. enterica*. The diversity of the prophage sequences described here was found to be sufficient in differentiating among *S. enterica* serovars, between genetically related isolates from different epidemiological events and between *Salmonella* and *Citrobacter, Hafnia*, and *Proteus*. Our data reveal a high diversity among *S. enterica* prophages which is herein applied to develop a novel subtyping tool for identifying *S. enterica* clusters.

## Author contributions

WM developed the prophage sequence analyses pipeline, conducted the data analyses, prepared the figures and tables and drafted the manuscript; M-OD wrote the pipeline script for prophage sequence analyses; AD, VU, and HH contributed to data analyses; JJ, LF, J-GE-R, JH, IK-I, BB, and RL developed the whole genome sequences of all the bacterial isolates, developed, curated, and maintained the SalFos database; AG, EB, EF, GA, JTW, SG, MW, FD, SM, SB, and LG provided bacterial isolates for whole genome sequencing and provided metadata; SB, RL, and LG were involved in the study design, data analysis and review; DO conceived the study and participated in data analysis, development of manuscript and review. All the authors revised and approved the manuscript.

### Conflict of interest statement

The authors declare that the research was conducted in the absence of any commercial or financial relationships that could be construed as a potential conflict of interest.

## References

[B1] Adriaenssens E. M. Cowan D. A. (2014). Using signature genes as tools to assess environmental viral ecology and diversity. Appl. Environ. Microbiol. 80, 4470–4480. 10.1128/AEM.00878-14 24837394 PMC4148782

[B2] Allard M. W. Luo Y. Strain E. Pettengill J. Timme R. Wang C.. (2013). On the evolutionary history, population genetics and diversity among isolates of *Salmonella* Enteritidis PFGE pattern JEGX01.0004. PLoS ONE 8:e55254. 10.1371/journal.pone.0055254 23383127 PMC3559427

[B3] Altschul S. F. Gish W. Miller W. Myers E. W. Lipman D. J. (1990). Basic local alignment search tool. J. Mol. Biol. 215, 403–410. 10.1016/S0022-2836(05)80360-2 2231712

[B4] Arndt D. Grant J. R. Marcu A. Sajed T. Pon A. Liang Y.. (2016). PHASTER: a better, faster version of the PHAST phage search tool. Nucleic Acids Res. 44, W16–W21. 10.1093/nar/gkw387 27141966 PMC4987931

[B5] Ashton P. M. Nair S. Peters T. M. Bale J. A. Powell D. G. Painset A.. (2016). Identification of *Salmonella* for public health surveillance using whole genome sequencing. PeerJ 4:e1752. 10.7717/peerj.1752 27069781 PMC4824889

[B6] Ashton P. M. Owen S. V. Kaindama L. Rowe W. P. M. Lane C. R. Larkin L.. (2017). Public health surveillance in the UK revolutionises our understanding of the invasive *Salmonella* Typhimurium epidemic in Africa. Genome Med. 9:92. 10.1186/s13073-017-0480-7 29084588 PMC5663059

[B7] Assis F. E. A. Dallagassa C. B. Farah S. M. S. S. Souza E. M. Pedrosa F. O. Chubatsu L. S.. (2017). Molecular characterisation of *Salmonella* strains isolated from outbreaks and sporadic cases of diarrhoea occurred in Parana State, South of Brazil. Epidemiol. Infect 145, 1953–1960. 10.1017/S0950268817000619 28367777 PMC9203290

[B8] Bankevich A. Nurk S. Antipov D. Gurevich A. A. Dvorkin M. Kulikov A. S.. (2012). SPAdes: a new genome assembly algorithm and its applications to single-cell sequencing. J. Comput. Biol. 19, 455–477. 10.1089/cmb.2012.0021 22506599 PMC3342519

[B9] Bekal S. Berry C. Reimer A. R. Van Domselaar G. Beaudry G. Fournier E.. (2016). Usefulness of high-quality core genome single-nucleotide variant analysis for subtyping the highly clonal and the most prevalent *Salmonella enterica* serovar Heidelberg clone in the context of outbreak investigations. J. Clin. Microbiol. 54, 289–295. 10.1128/JCM.02200-15 26582830 PMC4733192

[B10] Bobay L. M. Rocha E. P. Touchon M. (2013). The adaptation of temperate bacteriophages to their host genomes. Mol. Biol. Evol. 30, 737–751. 10.1093/molbev/mss279 23243039 PMC3603311

[B11] Boyd E. F. Carpenter M. R. Chowdhury N. (2012). Mobile effector proteins on phage genomes. Bacteriophage 2, 139–148. 10.4161/bact.21658 23275865 PMC3530523

[B12] Brüssow H. Canchaya C. Hardt W. D. (2004). Phages and the evolution of bacterial pathogens: from genomic rearrangements to lysogenic conversion. Microbiol. Mol. Biol. Rev. 68, 560–602. 10.1128/MMBR.68.3.560-602.2004 15353570 PMC515249

[B13] Caporaso J. G. Kuczynski J. Stombaugh J. Bittinger K. Bushman F. D. Costello E. K.. (2010). QIIME allows analysis of high-throughput community sequencing data. Nat. Methods 7, 335–336. 10.1038/nmeth.f.303 20383131 PMC3156573

[B14] Coil D. Jospin G. Darling A. E. (2015). A5-miseq: an updated pipeline to assemble microbial genomes from Illumina MiSeq data. Bioinformatics 31, 587–589. 10.1093/bioinformatics/btu661 25338718

[B15] Colavecchio A. Cadieux B. Lo A. Goodridge L. D. (2017b). Bacteriophages contribute to the spread of antibiotic resistance genes among foodborne pathogens of the enterobacteriaceae family - A review. Front. Microbiol. 8:1108. 10.3389/fmicb.2017.01108 28676794 PMC5476706

[B16] Colavecchio A. D'Souza Y. Tompkins E. Jeukens J. Freschi L. Emond-Rheault J. G.. (2017a). Prophage integrase typing is a useful indicator of genomic diversity in *Salmonella enterica*. Front. Microbiol. 8:1283. 10.3389/fmicb.2017.01283 28740489 PMC5502288

[B17] Cooke F. J. Wain J. Fookes M. Ivens A. Thomson N. Brown D. J.. (2007). Prophage sequences defining hot spots of genome variation in *Salmonella enterica* serovar Typhimurium can be used to discriminate between field isolates. J. Clin. Microbiol. 45, 2590–2598. 10.1128/JCM.00729-07 17522270 PMC1951247

[B18] Davies E. V. Winstanley C. Fothergill J. L. James C. E. (2016). The role of temperate bacteriophages in bacterial infection. FEMS Microbiol. Lett. 363:fnw015. 10.1093/femsle/fnw015 26825679

[B19] Diard M. Bakkeren E. Cornuault J. K. Moor K. Hausmann A. Sellin M. E.. (2017). Inflammation boosts bacteriophage transfer between *Salmonella* spp. Science 355, 1211–1215. 10.1126/science.aaf8451 28302859

[B20] Emond-Rheault J. G. Jeukens J. Freschi L. Kukavica-Ibrulj I. Boyle B. Dupont M. J.. (2017). A Syst-OMICS approach to ensuring food safety and reducing the economic burden of salmonellosis. Front. Microbiol. 8:996. 10.3389/fmicb.2017.00996 28626454 PMC5454079

[B21] Fang S. B. Tseng W. Y. Lee H. C. Tsai C. K. Huang J. T. Hou S. Y. (2009). Identification of *Salmonella* using colony-print and detection with antibody-coated gold nanoparticles. J. Microbiol. Methods 77, 225–228. 10.1016/j.mimet.2009.02.008 19236895

[B22] Feasey N. A. Dougan G. Kingsley R. A. Heyderman R. S. Gordon M. A. (2012). Invasive non-typhoidal salmonella disease: an emerging and neglected tropical disease in Africa. Lancet 379, 2489–2499. 10.1016/S0140-6736(11)61752-2 22587967 PMC3402672

[B23] Feasey N. A. Hadfield J. Keddy K. H. Dallman T. J. Jacobs J. Deng X.. (2016). Distinct *Salmonella* Enteritidis lineages associated with enterocolitis in high-income settings and invasive disease in low-income settings. Nat. Genet. 48, 1211–1217 10.1038/ng.3644 27548315 PMC5047355

[B24] Figueroa-Bossi N. Bossi L. (1999). Inducible prophages contribute to *Salmonella* virulence in mice. Mol. Microbiol. 33, 167–176. 10.1046/j.1365-2958.1999.01461.x 10411733

[B25] Figueroa-Bossi N. Uzzau S. Maloriol D. Bossi L. (2001). Variable assortment of prophages provides a transferable repertoire of pathogenic determinants in *Salmonella*. Mol. Microbiol. 39, 260–271. 10.1046/j.1365-2958.2001.02234.x 11136448

[B26] Fu L. Niu B. Zhu Z. Wu S. Li W. (2012). CD-HIT: accelerated for clustering the next-generation sequencing data. Bioinformatics 28, 3150–3152. 10.1093/bioinformatics/bts565 23060610 PMC3516142

[B27] Grimont P. Weill F. X. (2007). Antigenic Formulae of the Salmonella Serovars, 9th Edn Geneva: WHO Collaborating Centre for Reference and Research on Salmonella Available online at: https://www.pasteur.fr/sites/default/files/veng_0.pdf

[B28] Gurevich A. Saveliev V. Vyahhi N. Tesler G. (2013). QUAST: quality assessment tool for genome assemblies. Bioinformatics 29:1072–1075. 10.1093/bioinformatics/btt086 23422339 PMC3624806

[B29] Hiley L. Fang N. X. Micalizzi G. R. Bates J. (2014). Distribution of Gifsy-3 and of variants of ST64B and Gifsy-1 prophages amongst *Salmonella enterica* Serovar Typhimurium isolates: evidence that combinations of prophages promote clonality. PLoS ONE 9:e86203. 10.1371/journal.pone.0086203 24475087 PMC3901673

[B30] Jacobsen A. Hendriksen R. S. Aaresturp F. M. Ussery D. W. Friis C. (2011). The *Salmonella enterica* pan-genome. Microb. Ecol. 62, 487–504. 10.1007/s00248-011-9880-1 21643699 PMC3175032

[B31] Katoh K. Standley D. M. (2013). MAFFT multiple sequence alignment software version 7, Improvements in performance and usability. Mol. Biol. Evol. 30, 772–780. 10.1093/molbev/mst010 23329690 PMC3603318

[B32] Kingsley R. A. Msefula C. L. Thomson N. R. Kariuki S. Holt K. E. Gordon M. A.. (2009). Epidemic multiple drug resistant *Salmonella* Typhimurium causing invasive disease in sub-Saharan Africa have a distinct genotype. Genome Res. 19, 2279–2287. 10.1101/gr.091017.109 19901036 PMC2792184

[B33] Letunic I. Bork P. (2011). Interactive tree of life v2: online annotation and display of phylogenetic trees made easy. Nucleic Acids Res. 39, W475–W478. 10.1093/nar/gkr201 21470960 PMC3125724

[B34] Margot H. Stephan R. Guarino S. Jagadeesan B. Chilton D. O'Mahony E.. (2013). Inclusivity, exclusivity and limit of detection of commercially available real-time PCR assays for the detection of *Salmonella*. Int. J. Food Microbiol. 165, 221–226. 10.1016/j.ijfoodmicro.2013.05.012 23800733

[B35] Mmolawa P. T. Schmieger H. Heuzenroeder M. W. (2003). Bacteriophage ST64B, a genetic mosaic of genes from diverse sources isolated from *Salmonella enterica* serovar typhimurium DT 64. J. Bacteriol. 185, 6481–6485. 10.1128/JB.185.21.6481-6485.2003 14563886 PMC219385

[B36] Mottawea W. Chiang C. K. Mühlbauer M. Starr A. E. Butcher J. Abujamel T.. (2016). Altered intestinal microbiota-host mitochondria crosstalk in new onset Crohn's disease. Nat. Commun. 7:13419. 10.1038/ncomms13419 27876802 PMC5122959

[B37] Muldoon M. T. Teaney G. Li J. Onisk D. V. Stave J. W. (2007). Bacteriophage-based enrichment coupled to immunochromatographic strip-based detection for the determination of *Salmonella* in meat and poultry. J. Food Prot. 70, 2235–2242. 10.4315/0362-028X-70.10.2235 17969603

[B38] Ogunremi D. (2013). Strategy for developing a molecular subtyping tool for a foodborne bacterial pathogen using a whole genome analysis: the case of *Salmonella* Enteritidis, in 63rd Annual Conference of the Canadian Society of Microbiologists (Ottawa, ON), 199.

[B39] Ogunremi D. Devenish J. Amoako K. Kelly H. Dupras A. A. Belanger S. (2014). High resolution assembly and characterization of genomes of Canadian isolates of *Salmonella* Enteritidis. BMC Genomics 15:713 10.1186/1471-2164-15-713 25156331 PMC4165908

[B40] Ogunremi D. Kelly H. Dupras A. A. Belanger S. Devenish J. (2014). Development of a new molecular subtyping tool for *Salmonella enterica* serovar Enteritidis based on single nucleotide polymorphism genotyping using PCR. J. Clin. Microbiol. 52, 4275–4285. 10.1128/JCM.01410-14 25297333 PMC4313298

[B41] Okoro C. K. Barquist L. Connor T. R. Harris S. R. Clare S. Stevens M. P. (2015). Signatures of adaptation in human invasive *Salmonella* Typhimurium ST313 populations from sub-Saharan Africa. PLoS Negl. Trop. Dis. 9:e0003611 10.1371/journal.pntd.0003611 25803844 PMC4372345

[B42] Omer S. Harlow T. J. Gogarten J. P. (2017). Does sequence conservation provide evidence for biological function? Trends Microbiol. 25, 11–18. 10.1016/j.tim.2016.09.010 27773523

[B43] Owen S. V. Wenner N. Canals R. Makumi A. Hammarlöf D. L. Gordon M. A.. (2017). Characterization of the prophage repertoire of African *Salmonella* Typhimurium ST313 reveals high levels of spontaneous induction of novel phage BTP1. Front. Microbiol. 8:235. 10.3389/fmicb.2017.00235 28280485 PMC5322425

[B44] Public Health Agency of Canada [PHAC] (2012). In: *National Enteric Surveillance Program - Annual Summary*. Available online at: http://publications.gc.ca/collections/collection_2014/aspc-phac/HP37-15-2012-eng.pdf

[B45] Rohwer F. Edwards R. (2002). The phage proteomic tree: a genome-based taxonomy for phage. J. Bacteriol. 184, 4529–4535. 10.1128/JB.184.16.4529-4535.2002 12142423 PMC135240

[B46] Thomson N. R. Clayton D. J. Windhorst D. Vernikos G. Davidson S. Churcher C.. (2008). Comparative genome analysis of Salmonella Enteritidis PT4 and *Salmonella* Gallinarum 287/91 provides insights into evolutionary and host adaptation pathways. Genome Res. 18, 1624–1637. 10.1101/gr.077404.108 18583645 PMC2556274

[B47] Tritt A. Eisen J. A. Facciotti M. T. Darling A. E. (2012). An integrated pipeline for de novo assembly of microbial genomes. PLoS ONE 7:e42304. 10.1371/journal.pone.0042304 23028432 PMC3441570

[B48] WHO (2016) WHO Estimates of the Global Burden of Foodborne Diseases. Foodborne Disease Burden Epidemiology Reference Group 2007-2015. Available online at: http://www.who.int/foodsafety/areas_work/foodborne-diseases/ferg/en/

[B49] Zheng J. Luo Y. Reed E. Bell R. Brown E. W. Hoffmann M. (2017). Whole-genome comparative analysis of *Salmonella enterica* serovar Newport strains reveals lineage-specific divergence. Genome Biol. Evol. 94, 1047–1050. 10.1093/gbe/evx065 PMC5405337 28379364

[B50] Zhou Y. Liang Y. Lynch K. H. Dennis J. J. Wishart D. S. (2011). PHAST: a fast phage search tool. Nucleic Acids Res. 39, W347–W352. 10.1093/nar/gkr485 21672955 PMC3125810

[B51] Ziebell K. Chui L. King R. Johnson S. Boerlin P. Johnson R. P. (2017). Subtyping of Canadian isolates of *Salmonella* Enteritidis using Multiple Locus Variable Number Tandem Repeat Analysis (MLVA) alone and in combination with Pulsed-Field Gel Electrophoresis (PFGE) and phage typing. J. Microbiol. Methods 139, 29–36. 10.1016/j.mimet.2017.04.012 28456552

