# Supplementary figures and images for: Salmonella enterica Prophage Sequence Profiles Reflect Genome Diversity and Can Be Used for High Discrimination Subtyping
Source: Front Microbiol. 2018 May 4;9:836. doi: 10.3389/fmicb.2018.00836 (PMC5945981; doi:10.3389/fmicb.2018.00836)

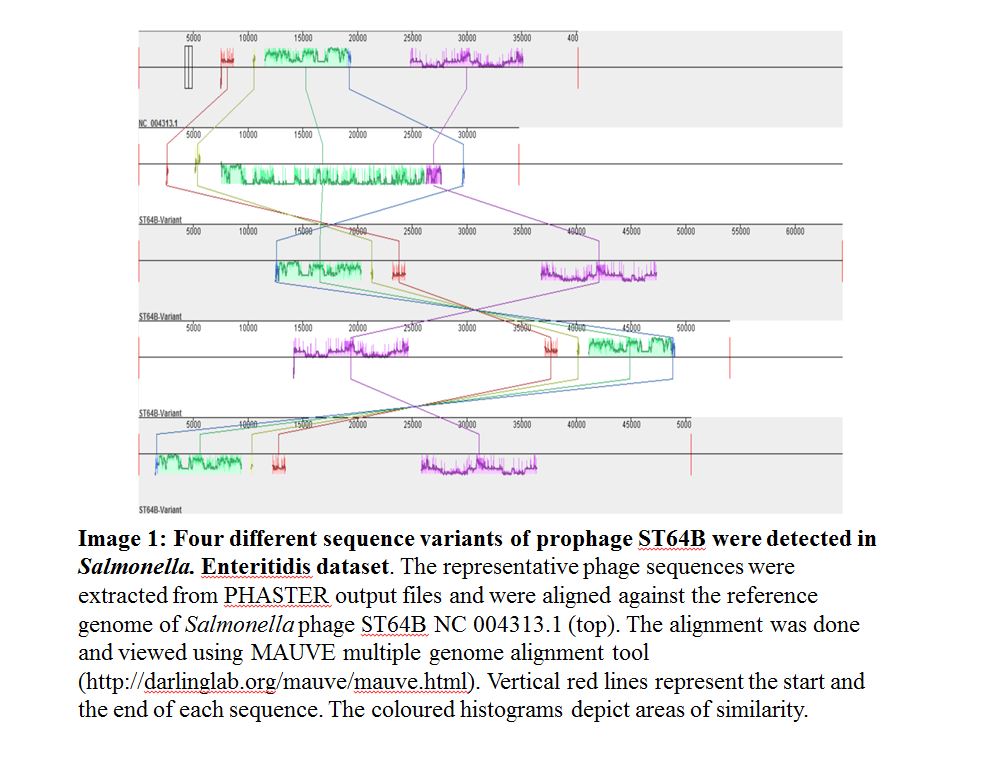

Supplement: Supplementary file 1 [file Image_1.jpg]

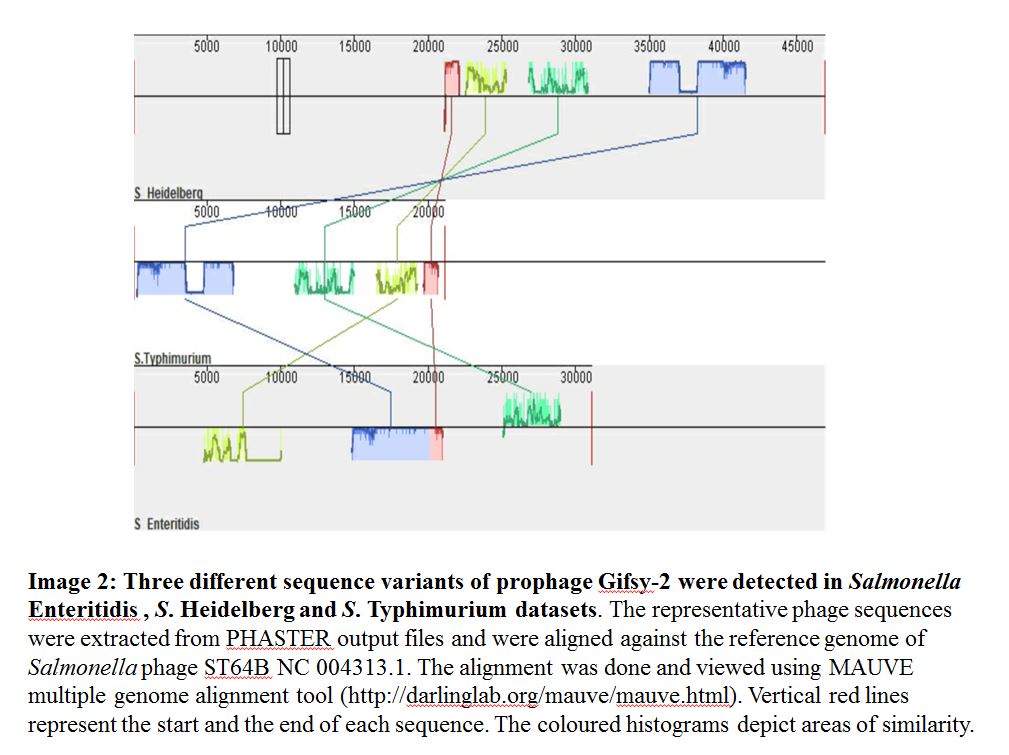

Supplement: Supplementary file 2 [file Image_2.jpg]
